# The Effect of Dexamethasone and Triiodothyronine on Terminal Differentiation of Primary Bovine Chondrocytes and Chondrogenically Differentiated Mesenchymal Stem Cells

**DOI:** 10.1371/journal.pone.0072973

**Published:** 2013-08-16

**Authors:** Thomas M. Randau, Frank A. Schildberg, Mauro Alini, Matthias D. Wimmer, El-Mustapha Haddouti, Sascha Gravius, Keita Ito, Martin J. Stoddart

**Affiliations:** 1 Department of Orthopedics and Trauma Surgery, University Clinic of Bonn, Bonn, Germany; 2 Institute of Molecular Medicine and Experimental Immunology, University of Bonn, Bonn, Germany; 3 AO Research Institute, AO Foundation, Davos, Switzerland; 4 Institute of Materials Technology, Eindhoven University of Technology, Eindhoven, Netherlands; Department of Biomaterials, Japan

## Abstract

The newly evolved field of regenerative medicine is offering solutions in the treatment of bone or cartilage loss and deficiency. Mesenchymal stem cells, as well as articular chondrocytes, are potential cells for the generation of bone or cartilage. The natural mechanism of bone formation is that of endochondral ossification, regulated, among other factors, through the hormones dexamethasone and triiodothyronine. We investigated the effects of these hormones on articular chondrocytes and chondrogenically differentiated mesenchymal stem cells, hypothesizing that these hormones would induce terminal differentiation, with chondrocytes and differentiated stem cells being similar in their response. Using a 3D-alginate cell culture model, bovine chondrocytes and chondrogenically differentiated stem cells were cultured in presence of triiodothyronine or dexamethasone, and cell proliferation and extracellular matrix production were investigated. Collagen mRNA expression was measured by real-time PCR. Col X mRNA and alkaline phosphatase were monitored as markers of terminal differentiation, a prerequisite of endochondral ossification. The alginate culture system worked well, both for the culture of chondrocytes and for the chondrogenic differentiation of mesenchymal stem cells. Dexamethasone led to an increase in glycosaminoglycan production. Triiodothyronine increased the total collagen production only in chondrocytes, where it also induced signs of terminal differentiation, increasing both collagen X mRNA and alkaline phosphatase activity. Dexamethasone induced terminal differentiation in the differentiated stem cells. The immature articular chondrocytes used in this study seem to be able to undergo terminal differentiation, pointing to their possible role in the onset of degenerative osteoarthritis, as well as their potential for a cell source in bone tissue engineering. When chondrocyte-like cells, after their differentiation, can indeed be moved on towards terminal differentiation, they can be used to generate a model of endochondral ossification, but this limitation must be kept in mind when using them in cartilage tissue engineering application.

## Introduction

The two major areas of musculoskeletal medicine are trauma care and degenerative disorders. Degenerative joint diseases account for half of all chronic conditions in people aged over 65. In young adults, it is foremost traffic accidents that produce a tremendous demand for restorative medical attention. It is estimated that traffic injuries worldwide result in more than 30 million severe or disabling injuries, with associated costs of 500 billion US dollars annually [1]. The newly evolved field of regenerative medicine is offering new solutions in treating problems arising from loss and deficiency especially of cartilage and bone [2].

Bone marrow-derived mesenchymal stem cells (MSCs) possess a number of abilities, making them interesting candidate cells for tissue engineering applications [3]. They have the capacity to differentiate into cells of connective tissue lineages, including bone, fat, cartilage and muscle. They can be isolated and expanded with high efficiency, and induced to differentiate to multiple lineages under defined culture conditions [4], but no routinely available clinical therapy has been generated from this approach.

In 1994, the transplantation of *ex vivo* expanded articular chondrocytes into cartilaginous defects of the knee joint was one of the first successful applications of tissue engineering [5]. Introduced into clinical practice more than two decades ago, the process known as autologous chondrocyte implantation (ACI) is now established, but is not free of controversies [6]. A dedifferentiation of the implanted chondrocytes towards a more fibrous cartilage as well as a terminal differentiation with calcification can occur in the implants, both resulting in an inferior joint surface. Scientific attention has therefore turned towards the use of MSCs for cartilage regeneration [7], but the phenotypic stability of the differentiated MSCs remains a major concern [8].

Another example for the potential use of tissue engineering in musculoskeletal medicine is the management of large bone defects which still pose a considerable challenge in musculoskeletal surgery. Other than autologous bone grafting, either in a local fashion or through bypass grafting or bone transport, there is no other effective method for management of diaphyseal defects [9]. It is already possible to create stable, osteoconductive, biocompatible and biodegradable implants, which may release osteoinductive molecules and which may be seeded with potentially osteogenic cells [10]. Vascularization is essential to bone regeneration and it is generally believed that a limited supply of nutrition due to a lack of blood supply in larger scaffolds is responsible for the lack of cell survival. Although diffusion may be sufficient for cells near the surface, neovascularization is too slow to supply the cells embedded deep within a graft, resulting in graft failure and non-unions. MSCs require a relatively nutrient rich environment (including oxygen) for survival [11], therefore the use of articular chondrocytes instead might offer an alternative. Cartilage is an avascular tissue, therefore chondrocytes have adapted to low oxygen tension and glycolysis for their metabolic needs [12].

Cartilage is routinely transformed into bone in in the process of endochondral ossification, during long bone growth and in fracture healing. Here, chondrocytes undergo a terminal step of differentiation, increasing in size and finally undergoing cell death by apoptosis, leaving behind a suitable matrix for bone ingrowth, vascularization and mineralization. This process was initially described in growth plate chondrocytes [13] and the fibrous cartilage of the soft fracture callus, but articular chondrocytes do seem to keep their abilities associated with skeletal development and bone growth. Expression of collagen type X (Col X), known to be a marker of chondrocyte terminal differentiation, has been seen in osteoarthritic chondrocytes [14], and was recently also shown to be involved in other degenerative diseases like intervertebral disc degeneration [15]. Common regulatory pathways and cytokines of regenerative and degenerative processes have recently been identified [16]. Among them are also the thyroid hormone triiodothyronine (T_3_) and the glucocorticoid dexamethasone (Dex) [17].

Though glucocorticoids (GCs) are frequently used *in vivo* and *in vitro* on cartilage and chondrocytes, their effect on this tissue is not yet fully understood, and their role in induction or prevention of endochondral ossification remains ambiguous. GCs signal largely through the GC receptor, a member of the nuclear receptor family that translocates into the nucleus upon ligand binding and acts as transcription factor [18], but the molecular targets of GCs in chondrocytes are largely unknown. Dex has a potent anti-inflammatory property and is used in treatment of a wide variety of inflammatory conditions such as rheumatoid arthritis. It has also been shown that Dex has a protective effect against degradation of cartilage. The anabolic and anti-inflammatory effect leaves GCs well established in clinical use [19]. Also Dex seems to be crucial for chondrogenesis of MSCs *in vitro* [20]. Extensive use of glucocorticoids in humans and animals is on the other hand well known to impair longitudinal growth. It has been shown that this effect might be caused by induction of apoptosis in chondrocytes of the growth plate [21] as well as chondrocytes [22].

Triiodothyronine, the deiodinated and thereby activated form of the thyroid hormone, might well be the most important growth hormone. It affects virtually every cell in the organism by binding to its intranuclear receptors T_3_-Receptor alpha (TRα) and T_3_-Receptor beta (TRβ), modifying transcriptional activity on the genome [23]. Its role in the regulation of long bone growth and endochondral ossification is crucial [24]. Congenital defects in the hormone or its receptor lead to dwarfism and retarded development [25]. *In vitro*, thyroid hormones have been frequently used to induce hypertrophy in fetal and growth plate chondrocytes in various culture systems [26–28]. Very little research has been conducted in the effect of T_3_ on adult articular cartilage, but it’s possible role in the development of OA has been suggested previously [29].

In this study, we investigated the effects of the two hormones T_3_ and Dex on primary articular chondrocytes and differentiated chondrogenic MSCs, using serum-free induction and the alginate bead three-dimensional (3D) cell culture model. The study was performed on material of a large animal model (bovine), so the results can be used for later establishment of *in vivo* models for cartilage degradation or bone tissue engineering constructs with some biomechanical validity. In addition to cell survival and matrix production, the experiments were designed to monitor phenotypic stability and furthermore to detect signs of terminal chondrocyte differentiation as a preliminary phase of endochondral ossification and mineralization. We hypothesized that both hormones will induce terminal differentiation in adult articular chondrocytes and chondrocyte-like cells. Furthermore, we expected articular chondrocytes and chondrogenically differentiated MSCs to be similar in their response to the hormonal stimuli.

## Materials and Methods

### Cell culture

Chondrocytes were isolated from articular cartilage of bovine fetlock joints from three calves (two to six month of age) [30]. Cartilage slices were harvested and washed in Tyrode’s balanced salt solution (TBSS, Gibco) containing 10% Penicillin/Streptomycin (PenStrep), then pre-digested with 0.1% Pronase (Roche) in Dulbecco’s modified eagle medium (DMEM, Gibco) for 120 min. The pre-digested cartilage was washed and subsequently digested with 600 U/ml Collagenase II (Roche) in DMEM by stirring overnight. The digest was filtered through a 40 µm cell strainer (Falcon), and the cells were collected by centrifugation and encapsuled into alginate beads as described below.

MSCs were isolated from full bone marrow aspirate of two calves (four and six month of age) as described in the literature [31]. Bone marrow was aspirated from the tuber ischiadicum into sodium heparin immediately after slaughter. The bone marrow sample was washed, passed through a 40 µm cell strainer and full bone marrow was then plated in 300 cm^2^ culture flasks (TPP, Switzerland) in control medium consisting of DMEM low glucose (1g/L) with 1% PenStrep, 25 mM HEPES Buffer (Gibco), 3.7 mg/ml NaHCO_3_, and 10% FCS. After being cultured for four days, non-adherent cells were removed by changing the culture medium. In the following, the medium was changed every two to three days. Primary cultures were considered passage 0 (P0). Upon subconfluence, the cells were detached by using 0.25% trypsin (Gibco) and split in three for the next passage. Cells of passage P2 to P3 were encapsuled in the alginate beads and used for experiments.

Alginate is a natural carbohydrate polymer that is derived from brown algae. The monomers gelate upon contact with a divalent ion such as calcium, forming an elastic hydrogel. Removal of calcium through a chelator unlinks the monomers, thereby dissolving the hydrogel and releasing the cells without digestion or disruption [32]. It is for these attributes that alginate was early introduced for tissue engineering research, especially with chondrocytes [33], and its beneficial effect on the chondrocyte phenotype has recently been investigated systematically [34]. For the encapsulation of cells in alginate beads, the cells of each individual animal were pelleted and resuspended in sterile 0.9% NaCl solution containing 1.2% sodium alginate to a final concentration of 4*10^6^ cells/ml, and expressed through a 22-gauge needle in a dropwise fashion into a 102 mM calcium chloride (CaCl_2_) solution. After instantaneous gelation, the beads were allowed to polymerize for no more than 10 minutes, washed and cultured in 6-well plates, coated with 3% agarose, approximately 30 beads per well in 6 ml of medium [35].

For differentiation, alginate beads with encapsulated MSCs of passage 2 to 3 were grown in a standard chondrogenic medium, consisting of DMEM, supplemented with 1 µg/ml insulin, 1ng/ml transferrin and 1 ng/ml sodium selenite (ITS+1), 60 µg/ml ascorbic acid, 100 nM dexamethasone (all Sigma) and 10 ng/ml transforming growth factor beta-1 (TGF-β1, Sigma) for 3 weeks. Alginate beads without TGF-β1 were cultured as controls to evaluate the effect of the three dimensional culture on the cells. MSCs in monolayer, receiving standard expansion medium, were used as a negative control. Though the standard protocol for chondrogenic differentiation is the pellet culture system [36], alginate beads were used so that the cells could be submitted to the same protocol as the adult chondrocytes afterwards, but pellet cultures were performed as a positive control. For the pellet cultures, 2.5 * 10^5^ MSCs were resuspended in expansion medium and were spun down (10 min @ 500g) in a 15-ml conical tube. Pellets received standard chondrogenic medium as described above, with TGF-β1 [37]. Pellets were cultivated at 37°C in a humidified atmosphere including 5% CO_2_ with loosened caps for 20 days, changing the medium three times per week.

Alginate beads with cells were kept in agarose-coated 6-well tissue culture plastic dishes (TPP, Switzerland) in a cell culture incubator at standard conditions (37°C, 5% CO2, humidified atmosphere). The chondrocyte medium was composed of high glucose DMEM, supplemented with 1% Pen/Strep, 0.3% Gentamycin, 25mM HEPES Buffer, 60 µg/ml L-ascorbic acid 2-phosphate and 10% FCS. For hormonal treatment, serum was replaced by 1 mg/ml bovine serum albumin, 1 µg/ml insulin, 1ng/ml transferrin and 1 ng/ml sodium selenite, and the medium was also supplemented with 100nM (=10^-7^ M) triiodo-L-thyronine sodium salt (T_3_) in 10mM sodium hydroxide (NaOH, Fluka) or 1 µM Dex, respectively. Hormone concentrations were adapted from related studies in the literature to ensure comparability. Pilot experiments with lower concentrations of the hormones showed no significant effect of differences in concentration (data not shown).

**Figure 1:**
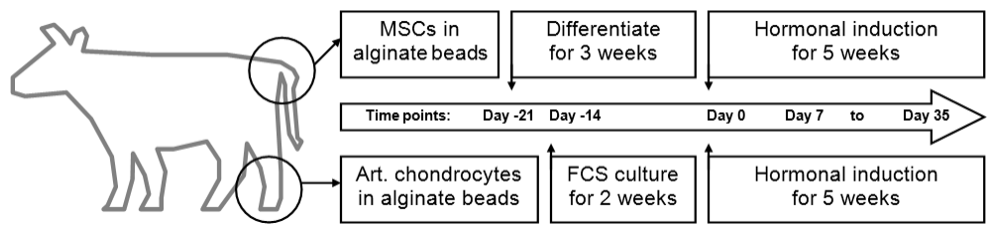
Experimental layout and timepoints. MSCs were harvested from the tuber ischiadicum and chondrocytes from fetlock joints, and both cells were encapsuled in alginate beads. While the MSCs were differentiated chondrogenically for three weeks (day -21 to day 0), the chondrocytes were kept in serum medium for two weeks (day -14 to day 0). The beads were then submitted to hormonal treatment for five weeks (day 0 to day 35) with weekly samples for analysis.

Before start of the hormonal treatment, chondrocyte beads were cultured in DMEM and in the presence of 10% FCS to allow for proliferation and matrix production for two weeks, and MSCs were differentiated as explained above for 3 weeks. The beads were then kept in hormone supplemented serum free culture for five weeks, with medium changes three times a week. Figure 1 illustrates the experimental layout and the timepoints.

To investigate calcification, 10 µM β-glyerophosphate (β-GP) was added to the medium of some beads on day 35. The samples were then cultured for a further 7 days and harvested.

### Biochemistry

Quantification of (DNA) was carried out using the Hoechst 33258 fluorescent dye as described elsewhere [38] with some modifications. Three samples consisting of three beads each were taken at each time point and from each group and stored frozen at -20°C until further analysis. For the assay, the beads were digested in 150 µl/bead (approximately 10 volumes) of a dissolution/digestion buffer, consisting of 150 mM NaCl (Fluka), 55 mM sodium citrate (Sigma), 5 mM disodium-EDTA (Sigma), 5 mM cysteine hydrochloride (Fluka) and 125 µg/ml Papain from Papaya latex (Sigma), dissolved in Millipore-filtered water. The beads were agitated gently, and incubated at 56°C for 15 hours. DNA was quantified using bisBenzimide Hoechst-33528 fluorescent dye (Fluka) at a concentration of 0.4 µg/ml in Dulbecco’s Phosphate Buffered Saline (DPBS). Calf Thymus DNA, diluted in the same digestion buffer as described above was used as standard. 40 µl of sample or standard were mixed with 160 µl of the dye in a white-bottom 96-well-plate (Falcon), incubated for 20 minutes, and fluorescence emission was read at 465 nm after excitation at 360 nm in a PerkinElmer HTS 7000 Bio Assay Reader. All measurements were done in duplicates, and DNA concentration was calculated from fluorescence emission using the linear range of the standard curve. All measurements were done in duplicates.

For hydroxyproline (HYP) detection, 250 µl of the digested sample were mixed with an equal volume of 12N HCl (Fluka) and hydrolyzed at 110°C for 12h. Samples were neutralized adding 300 µl of 10N NaOH (Fluka), cleared using a charcoal resin mix, pH was checked with indicator paper and titrated to neutrality with HCl. A standard curve was prepared from a stock solution of trans-4-hydroxy-L-proline (Fluka), and 250 µl of all samples and standards was transferred into a fresh 2 ml tube. All samples and standards were mixed with 250 µl of saturated NaCl. Oxidation of HYP was reached by addition of 500 µl of a pH 6-buffered Chloramine T solution, containing 120 mmol/l citric acid, 440 mmol/l sodium acetate, 425 mmol/l sodium hydroxide, 105 mmol/l acetic acid, 33 mmol/l Chloramine T trihydrate and 30% v/v 2-Methoxyethanol in water, mixing and leaving 20 minutes at room temperature. Color formation occurred after addition of 500 µl of a color solution, containing 11.25% w/v 4-(Dimethylamino)benzaldehyde, 2 mol/l perchloric acid and 50% v/v 1-Propanol in water, and incubating at 65°C for 15 minutes [39]. Absorbance was read in a PerkinElmer HTS 7000 Bio Assay Reader at 550 nm, and concentration of HYP was calculated from the standard curve. All measurements were done in duplicates.

Total sulfated proteoglycans (GAGs) were quantified as a marker of matrix synthesis in the alginate beads, using the dimethylmethylene blue assay as described elsewhere. The dye is known to give a positive reaction with alginate, which is constant over a broad range of values, and the problem can be circumvented by some modifications of the assay [40]. Samples prepared for the DNA assay were also used for measurement of total GAG. The dye solution was prepared from 16 µg/ml of 1,9- dimethyl-methylene blue (SigmaAldrich) in Milli-Q-filtered water, containing 0.03 M sodium formate (Sigma), 0.2% formic acid (Sigma) and 0.5% ethanol, pH 6.8. Chondroitin 4-sulfate sodium salt from bovine trachea (Fluka) was used as a standard, prepared as serial dilutions in a buffer containing 0.03% alginic acid sodium salt (Fluka), 0.05 M sodium acetate anhydrous (Fluka), 5.5 mM Na _3_Citrate (Sigma), 3 mM Na _2_EDTA (Sigma), 18.75 mM NaCl (Fluka) and 0.05% Tween 20 (Sigma). Samples were diluted in the same buffer to match the linear range of the standard curve. Dye was added to samples and standards in a 96-well culture plate (Falcon), after all samples and standards were mixed with Guanidine Hydrochloride solution to a final concentration of 240 mM. Absorbance was measured immediately at 535nm and 595nm in the Bio Assay Reader. Using the absorbance ratio, total amounts of GAG were calculated from the linear range of the standard curve. All measurements were done in duplicates.

Alkaline phosphatase (ALP) can be considered a marker of osteogenesis. Cells were released from alginate beads as described above with slight modifications. Three samples of three beads each were dissolved in a sterile dissolution buffer consisting of 150 mM NaCl (Fluka) and 55 mM Na _3_Citrate.2H_2_O (Sigma). Cells were collected by centrifugation (10 minutes at 700 G). The pellet was resuspended in a 10mM Tris-HCl buffered (pH 7.4) solution containing 0.1% Triton-X and extracted at 4°C under agitation for exactly 60 minutes. Samples were then centrifuged 10 minutes at 1000g to remove cells and cell debris, and the supernatant was transferred into a fresh tube. Samples were stored frozen at -20°C until analysis. Activity of ALP protein was measured by adding 50 µl of a substrate solution, containing 25 mg/ml of 4-nitrophenyl phosphate disodium salt hexahydrate (Sigma) in a 1M diethanolamine-buffered 0.5mM MgCl_2_ solution (pH 9.8), to 100 µl of sample in 250 µl of alkaline buffer containing 1.5 M 2-amino-2-methylpropanol (pH 10.3) and 100 µl of water, and incubating at 37°C for exactly 15 minutes. The reaction was stopped by addition of 500 µl of 0.1M NaOH. The amount of p-nitrophenol released was measured spectrophotometically at 405 nm, and amounts were calculated from a standard curve, prepared from a stock solution of p-nitrophenol (Sigma) in the same buffer [41].

### Histology

Methanol fixed alginate beads or pellets were transferred to a 30% sucrose solution overnight, containing 0.1 M CaCl_2_ to prevent the beads from dissolving. Beads were soaked in Cryocompound (Leica) and sectioned on a cryotome to sections of 8 µm and mounted on SuperFrost Plus slides. Sections were kept frozen until stained.

Alcian blue interacts with alginate but lowering the pH of the dye solution to 1.0 suppresses background staining of alginate and enables a selective staining of sulfated PGs only [32]. Slides were rehydrated and transferred to 3% acetic acid for 3 minutes, then stained in alcian blue dye (1% w/v Alcian blue 8GX, Fluka, in 3% acetic acid solution, containing 0.1 M CaCl_2_, pH adjusted to 1.0) for 30 minutes. Slides were washed in 0.1 M CaCl2, dehydrated and mounted with DPX water-free mountant (Sigma).

Though described in the literature [42,43] alizarin red (AR) staining in alginate beads has its difficulties, since the beads are gelated through the addition of CaCl_2_ solution, bound to cause considerable background staining. The slides were rehydrated and stained in a filtered AR-solution (2% Alizarin Red S, Fluka, pH adjusted to 4.2 with ammonia), blot dried, and dipped in acetone [44], then dehydrated in xylene and mounted as described above.

### Real Time RT-PCR

Three times ten beads per group and time point were dissolved in the sterile dissolution buffer described above and cells were collected by centrifugation (10 minutes at 700g). Total RNA was then extracted from the samples using TRI Reagent with poliacrylic carrier (Molecular Research Center), and RNA was purified using the Sigma GenElute Mammalian Total RNA Miniprep Kit (Sigma), according to manufacturer’s directions. Isolated mRNA was transcribed to complementary DNA (cDNA), using Applied Biosystems reverse transcription kit and a reaction of 25°C for 10 minutes, 48°C for 30 minutes, 95°C for five minutes on a thermo cycler (Eppendorf). The primers and probes (MicroSynth) used for the detection of collagen types I, II and X mRNA were custom designed and the sequences are listed in table 1. 18S ribosomal RNA (18S rRNA, Applied Biosystems) was used as endogenous control, and validation experiments for all primer-probe sets were performed on freshly isolated mRNA from the calcifying deep layer of articular cartilage from a bovine fetlock joint, as described elsewhere [45]. PCR was carried out using TaqMan PCR MasterMix (Applied Biosystems), with a concentration of 900 nM for the forward and reverse primers and 250 nM for the probes, and appropriate amounts of cDNA. The PCR was performed on an AB7500 system, running 2 minutes at 50°C, 10 minutes at 95°C followed by 40 cycles of 15 sec at 95°C and 1 min at 60°C for amplification, detecting fluorescence in Stage 3, Step 2. Data analysis was performed using the comparative C_T_ method, using 18S rRNA as endogenous control and the day 0 control-group as calibrator [46].

**Table 1:** Primer and probe sequences used for RT-PCR.

Gene	Primer fw 5’–3’	Primer rev 5’–3’	Probe 5’FAM/3’TAMRA
Procollagen 1A2 (bCol 1)	^149^ TGC AGT AAC TTC GTG CCT AGC A ^170^	^234^ CGC GTG GTC CTC TAT CTC CA ^215^	^172^ CAT GCC AAT CCT TAC AAG AGG CAA CTG C ^199^
Procollagen 2A1 (bCol 2)	^112^ AAG AAA CAC ATC TGG TTT GGA GAA A ^136^	^187^ TGG GAG CCA GGT TGT CAT C ^169^	^141^ CAA CGG TGG CTT CCA CTT CAG CTA TGG ^167^
Collagen X (bCol X)	^1923^ ACT TCT CTT ACC ACA TAC ACG TGA AAG ^1949^	^2030^ CCA GGT AGC CCT TGA TGT ACT CA ^2008^	^1985^ TGC CGT TCT TAT ACA GAC CTA CCC AAG CAT G ^1955^

Sequences of forward and reverse primers and probes used for the real time RT-PCR. Probes and primers were self-designed and supplied by MycroSynth (Switzerland) and prepared according to manufactures directions. Validation experiments on bovine articular chondrocytes and growth plate chondrocytes were carried out to verify equal efficiency.

### Data Management and Statistical Analysis

Raw data from measurement apparatus (Plate reader, Real-time PCR machine) was exported to CSV files, and imported into Microsoft Excel 2007 (Microsoft Corporation, Redmond, WA, USA). Data management, calculations from standard curves and dCt-Calculations for RT-PCR, as well as averaging of duplicate or triplicate measurements of the same sample was completed using Microsoft Excel 2010. Calculated and averaged data was then transferred to Graph Pad Prism 5.04 (GraphPad Software Inc., La Jolla, CA, USA) for statistical analysis. Statistics were completed using multifactor ANOVA, and p-values were calculated with Bonferroni post-hoc tests from the ANOVA, and are stated in the text and graphs. P < 0.05 was assumed “significant difference”. All analysis was completed with GraphPad Prism 5.04 for Windows, and all graphs were prepared with the same software. The authors received help with the statistical analysis by other persons as mentioned below (see acknowledgments). Unless stated otherwise, within the graphs, statistically significant difference between time points are indicated in asterisks as follows: *: p<0.05; **: p < 0.01; *** p < 0.001; ****: p < 0.0001. Statistically significant differences between treatment groups are indicated in crosses as follows: ^+^: p<0.05; ^+ +^: p < 0.01; ^+ + +^ p < 0.001; ^+ + + +^: p < 0.0001.

## Results

To compare primary chondrocytes to chondrogenically differentiated MSCs, the stem cells were differentiated in the alginate beads. During the differentiation, the cells acquired the properties of chondrocytes, including production of the cartilage-specific Col II and the production of a hyaline like extracellular matrix, composed of collagen and proteoglycans. Successful differentiation was therefore assumed with the expression of Col II mRNA and an increase in proteoglycan production.

**Figure 2:**
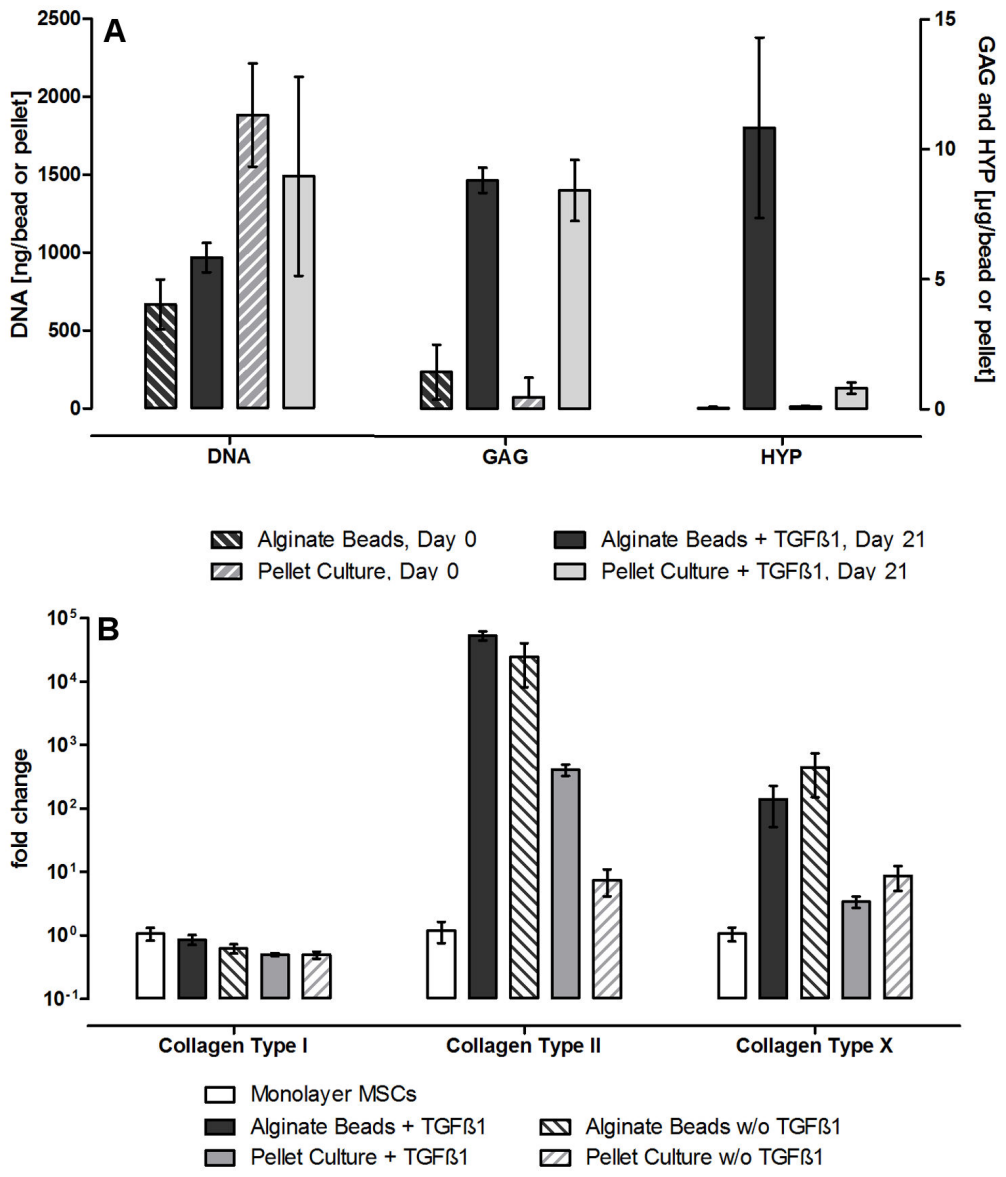
Production of DNA, GAG and HYP and collagen mRNA expression during chondrogenic MSC differentiation. The upper graph A shows the changes in total DNA content per bead (or per pellet, respectively) during the differentiation and the increase of proteoglycans and hydroxyproline as a sign of extra cellular matrix production. Both pellet cultures and alginate bead cultures show an increase in GAG, but only in alginate beads, also collagen is produced and retained. Shown is the mean of three samples per animal (n=6) +/- standard error of the mean (SEM). The lower graph B shows the fold-increase (mean +/- SD) of collagen mRNA, compared to undifferentiated MSCs kept in monolayer in expansion medium for 3 weeks. The bars compare alginate beads with and without TGF-β1 vs. pellet cultures with and without TGF-β1. Alginate beads with TGF-β1 show the highest increase in Col II mRNA expression, indicating that the culture system is well suited for chondrogenic differentiation of the MSCs.

MSCs in alginate beads proliferated only little, and at day 21, DNA content was increased by app. 30%, while in pellet culture the total DNA content decreased by 20% during the differentiation period (see Figure 2A). This decrease is associated with cell death in the center of the pellets, due to the lack of nutrients. The alginate bead hydrogel seems to bypass this problem, allowing nutrient supply even to the center of the bead. After establishing and testing the alginate bead cell culture system for the chondrogenic differentiation of mesenchymal stem cells, and for the culture of primary chondrocytes, the culture system was used to investigate whether or not T_3_ and Dex could influence chondrocyte differentiation towards hypertrophy and endochondral ossification. For this, the beads with chondrogenically differentiated MSCs, or serum precultured chondrocytes, were cultured in serum free medium containing either dexamethasone or T_3_ for five weeks

**Figure 3:**
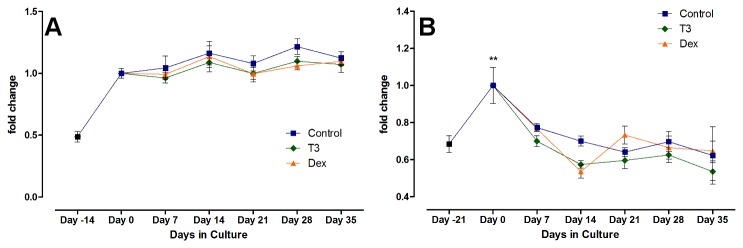
DNA content over time. Shown is the relative DNA content in articular chondrocytes (A), with day 0 being the reference and indicating the beginning of the hormonal treatment phase and day -14 showing the DNA content right after encapsulation. DNA content ranged from 96% to 121% of day 0 amounts, with no significant differences between treatment groups or over time. The second graph (B) shows the DNA content in the MSCs before and during differentiation (Day -21 to Day 0) and under the influence of T3 and Dex. The significant cell proliferation that occurred during the chondrogenic differentiation (Day 0 vs. Day 21, p<0.01) did not continue in the serum free treatment medium, indicating its dependence on TGF-β1.

No differences in DNA content were found between the chondrocyte treatment groups at any time point after “day 0”, which indicates the start of the hormonal treatment. In the MSCs, as described above, DNA content increased significantly during differentiation then dropped back to the levels of the initial seeding density within the first 7 days of hormonal treatment. No differences in DNA content were seen due to the hormonal treatment (see Figure 3).

The mRNA levels of Col I, II and X over 21 days of MSC chondrogenesis are shown in Figure 2B. Alginate beads with and without TGF-β1 and pellet cultures with TGF-β1, but not pellet cultures without TGF-β1 showed a significant increase in Col II mRNA expression over control. The changes in Col I mRNA and Col X mRNA remained statistically insignificant, but the alginate beads showed a trend towards higher expression of Col X mRNA as compared to the pellets. As it was expected, Collagen Type II surpassed Collagen Type I as the most commonly expressed collagen mRNA in both structures when cultured in the presence of TGF-β1.

**Figure 4:**
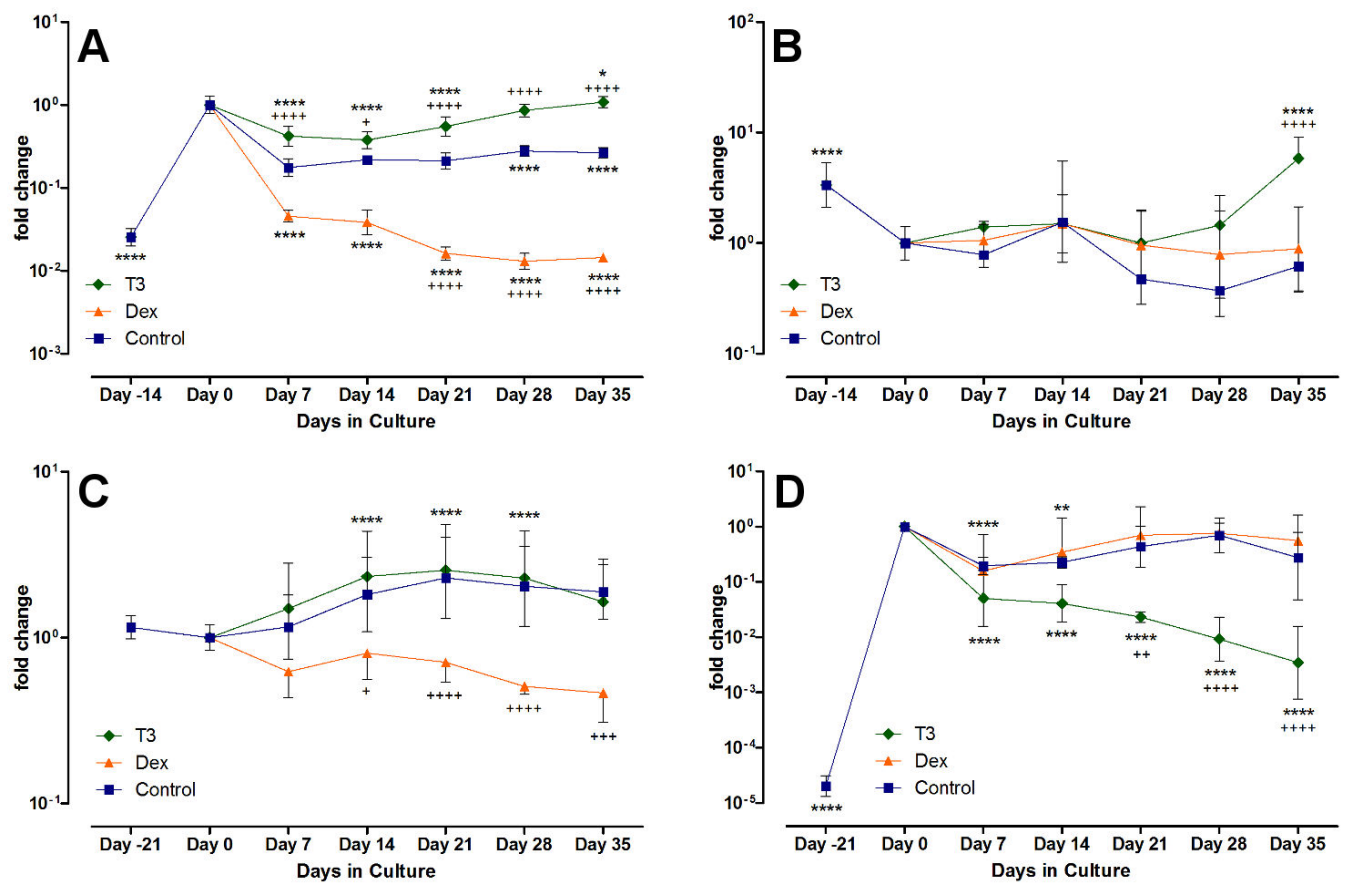
mRNA expression of Col I and Col II. Displayed is the relative change in Col type I and II mRNA in chondrocytes (A and B) under hormonal treatment vs. control, and in the MSCs (C and D, respectively). The expression was calibrated to 18S and normalized to control at day 0. Dex decreased Col I mRNA expression in both cell types significantly, but in greater scale in the chondrocytes (Dex vs. control with P < 0.001 after 21 days of culture). Col II mRNA was not significantly regulated by Dex, but decreased through T3 in MSCs and increased in chondrocytes.

In articular chondrocytes, T_3_ had an up-regulating or stabilizing effect on mRNA expression of Col I and Col II. Col II was not significantly modulated by Dex, but after five weeks of culture, the T_3_ treated group showed the highest Col II mRNA, even surpassing the amounts in freshly isolated chondrocytes (P < 0.0001 at day 35, T_3_ vs. Control). Col I, foremost a marker of de-differentiation in the articular chondrocytes, was significantly decreased by Dex, and no up regulation occurred during the whole observation period. Control and T_3_ group, in contrast, showed a steady state to slight increase, starting after two weeks of culture. With regard to Col I, Dex had a very similar decreasing effect on the chondrogenically differentiated MSCs as on adult chondrocytes (with P < 0.05 on day 14 vs. control, and P < 0.0001 thereafter). T_3_ had no effect significantly different than from the control group, and by day 35, Col I mRNA levels in T_3_ and control group were not significantly different than on day 0. For Col II, however, T_3_ significantly decreased mRNA expression as compared to control (P < 0.001 at day 21, and P < 0.0001 afterwards), while the levels remained mostly stable under control treatment or with Dex supplementation, thereby showing a reversed effect on the hormones when compared to adult chondrocytes (see Figure 4).

**Figure 5:**
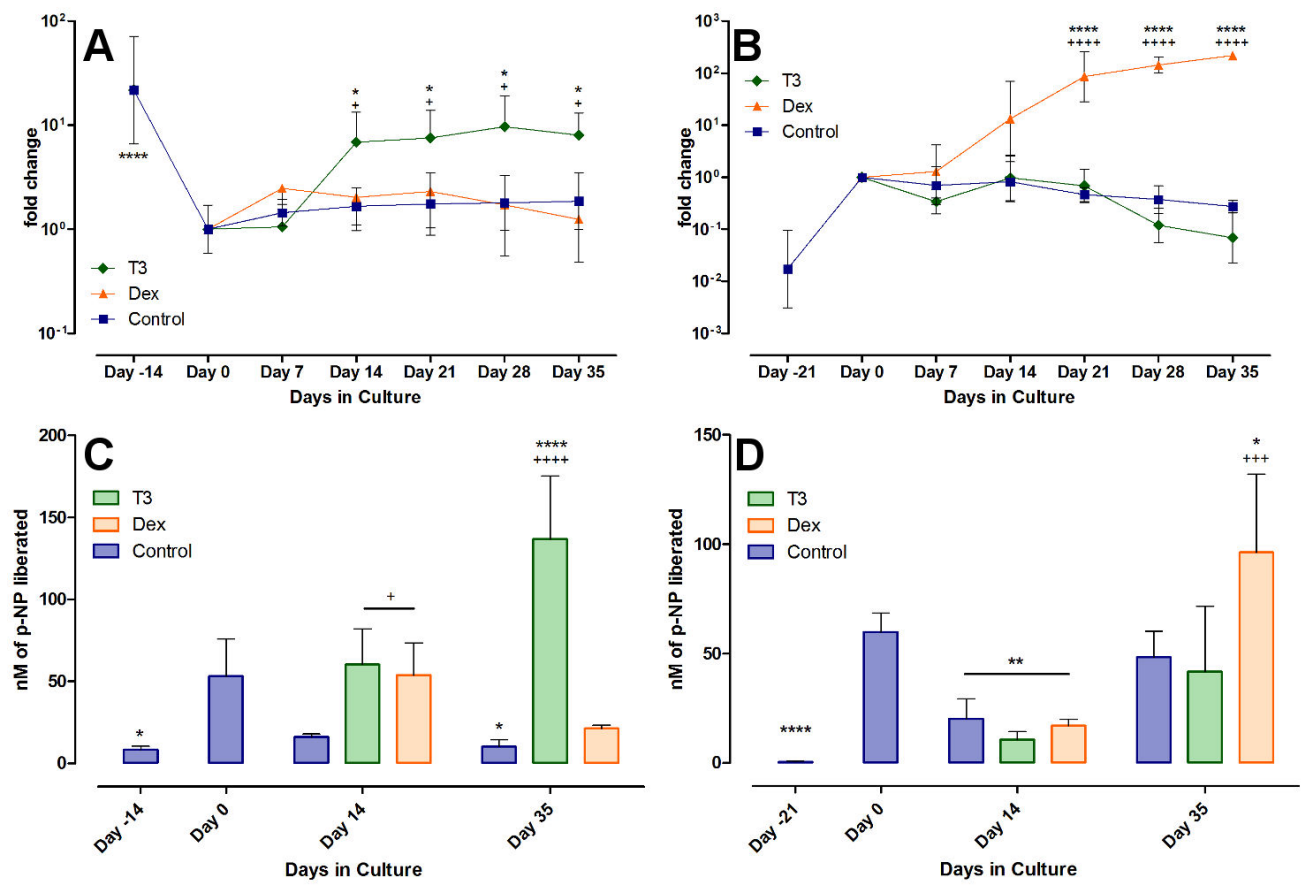
Markers of hypertrophy in chondrocyte and MSC culture. Shown is the relative change in Col type X mRNA in chondrocytes (A) and MSCs (B) and the total level of ALP activity (C and D, respectively). Both markers demonstrate that for articular chondrocytes, T3 can induce terminal differentiation in the cells, while chondrogenically differentiated MSCs show a similar response but to dexamethasone instead.

Col X mRNA expression and ALP protein activity were monitored over the culture period as markers of hypertrophy (Figure 5). Collagen Type X mRNA was found to be unexpectedly high in the freshly seeded chondrocyte beads. It rapidly decreased two to three orders of magnitude (P < 0.0001, day -14 vs. day 0) by week two or three, to then rise again only in the presence of T_3_. Control and Dex groups remained rather low, or increased only slowly. After 5 weeks of hormonal treatment, Collagen X levels were highest in the T_3_ treated groups, surpassing the other groups 10 to 20 fold and reaching up to 25% of the initial amounts at day 0. Initially rather low, the ALP activity was increased significantly during serum preculture (P < 0.05, day -14 vs. day 0), to then drop again when treated with control medium or Dex, with the drop quickest in the control group (P < 0.05, Dex and T_3_ vs. Control on day 14). Correlating to the increase in col X mRNA, ALP activity also went up in the T_3_-treated chondrocyte group (P < 0.0001, T_3_ vs. Control and significantly higher than at day 0, P < 0.0001).

The chondrogenically differentiated MSCs showed no change in Col X mRNA expression in response to T_3_ over the culture period of five weeks, as compared to control or over time. After the aforementioned increase during the differentiation, the levels of Col X mRNA remained stable, with a slight but non-significant tendency to decrease during culture with T_3_. In the MSCs, Dex had a significant impact, and increased Col X mRNA production significantly over time (P < 0.0001, from day 21 on) and against control group (P < 0.0001, Dex vs. control after 21 days). After five weeks, Col X mRNA levels were increased by two orders of magnitude compared to start of induction.

In contrast to articular chondrocytes, freshly isolated MSCs showed no measurable ALP protein activity. Expansion in monolayer did not change this. After encapsulation and chondrogenic differentiation, ALP activity became measurable and rose significantly to levels comparable to those of serum-cultured articular chondrocytes (Figure 5, P < 0.0001, day -21 vs. day 0). With removal of serum supplement and start of the induction, ALP activity dropped again in all three groups (P < 0.01, day 14 vs. day 0), and only with Dex was the activity after 35 days significantly higher than at day 0 (P < 0.05) and higher than in the control group (P < 0.001), correlating with the results from the Col X mRNA findings.

Proteoglycan production did not show much difference between the two groups, both in pellets and beads, cells produced significant amounts of glycosaminoglycan’s, as it is typical of chondrogenic cells.

Collagen, however, was either not produced in the pellets, or was not bound within and was lost during medium changes and washing. The alginate bead system, suspending the cells in a three dimensional hydrogel, seems to allow for a better deposition and binding of extra cellular matrix proteins.

**Figure 6:**
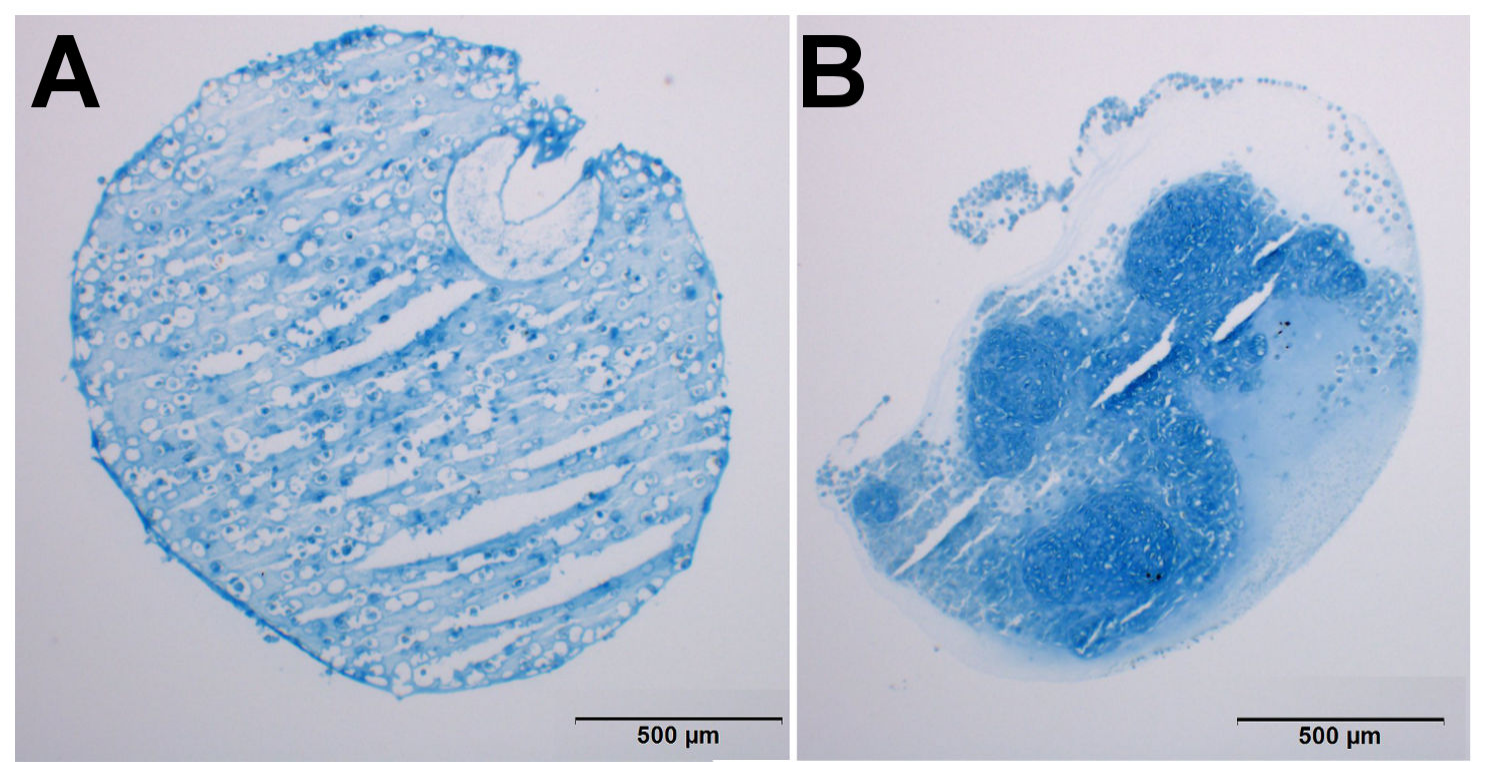
Stained cryosections of a pellet culture and an alginate bead. Alcian blue stain of 8 µm Cryosections of an alginate bead culture (A) and an MSC pellet culture (B) after 21 days of chondrogenic differentiation. Proteoglycans show in blue. The gaps in the alginate bead section are artifacts from the cutting. The scale bar represents 500 µm.

To evaluate the distribution of the cells within the beads and pellets, cryosections were stained for glycosaminoglycans with alcian blue. Cells could be seen distributed evenly throughout the alginate beads. Compared to the pellet cultures, alginate beads are larger in size and with a lower cell density; therefore the gaps between nuclei are much larger. Figure 6 illustrates the two culture systems, confirming the comparable amount of proteoglycans within the structures.

**Figure 7:**
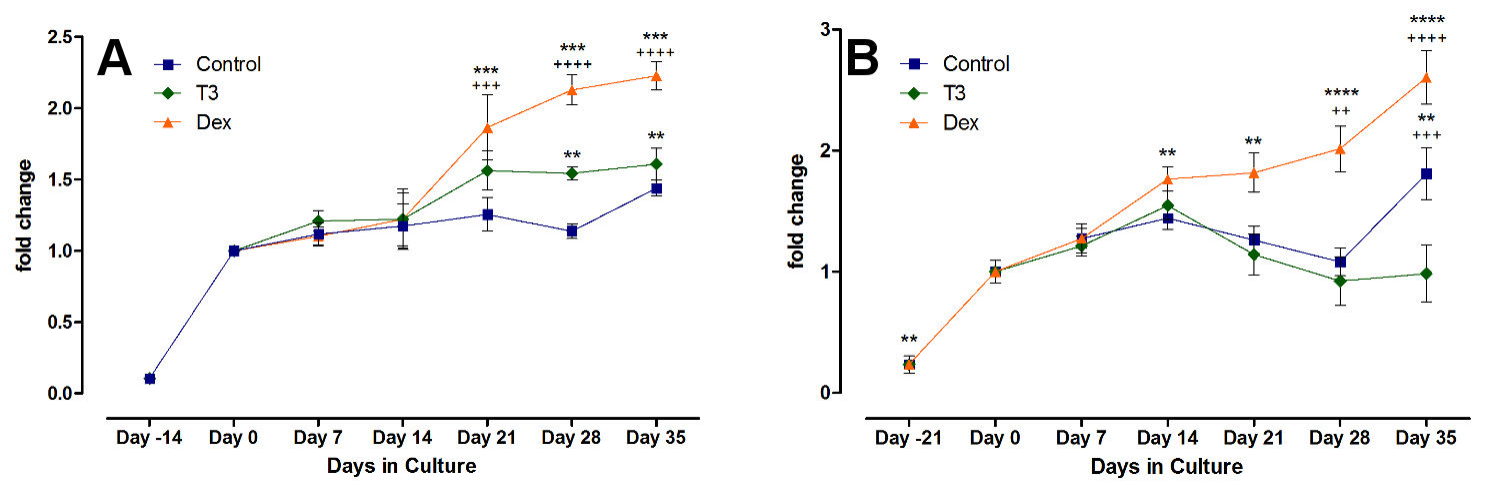
GAG content over time in the treatment groups. Shown is the relative GAG per DNA content of chondrocytes (A) and MSCs (B), with day 0 being the reference and indicating the beginning of the hormonal treatment. Absolute amounts of GAG reached more than 60 µg GAG/µg DNA or more than 80 µg of GAG per bead in the Dex group. After changing to serum-free hormonal treatment medium, Dex enhanced the GAG production significantly more than T3 or control medium (seen from day 21 on, with P < 0.001) and showed a general increase over time. A further increase with T3 after preculture serum showed only at day 28 (P < 0.01 vs. day 0), the control group had no significant increase in GAG/DNA after change to serum free medium. The MSCs behaved similar in their response to Dex, with significant increase in GAG production after two weeks (P < 0.01 at day 14 and 21 vs. day 0, and P < 0.0001 afterwards), but T3 seemed to rather decrease GAG production.

Proteoglycans are the major component of the cartilage ECM. The total proteoglycan content was analyzed from the same weekly samples as DNA content, and normalized to DNA amount to equalize for the differences in bead size and cell density. Any increase in GAGs can therefore be contributed to an increased synthesis per cell. Only the GAGs retained within the alginate beads were quantified. The results are displayed in Figure 7.

GAG content of freshly prepared chondrocyte beads after cell isolation and matrix digestion was low as expected and increased during serum preculture. Thereafter, only with Dex did the production increase further. The MSCs displayed a similar pattern as the articular chondrocytes. As stated above, the total amount of GAG had already increased during the chondrogenic differentiation period (see Figure 2A), comparable to the GAG production of the chondrocytes during the serum pretreatment phase. Subsequently, the GAG content increased significantly only in the Dex-treated group, as seen with articular chondrocytes. While articular chondrocytes displayed a slow increase in GAG/DNA in the T_3_-treated group, this effect was not visible with the MSCs. In contrast, the relative amount of GAG seemed to drop, though not significantly, from day 21 on. Without any hormonal supplements, in the control group, the GAG content was steady and showed significant increase at day 35 (P < 0.01 vs. day 0, and significantly higher with P < 0.001 vs. T_3_ at the same time).

**Figure 8:**
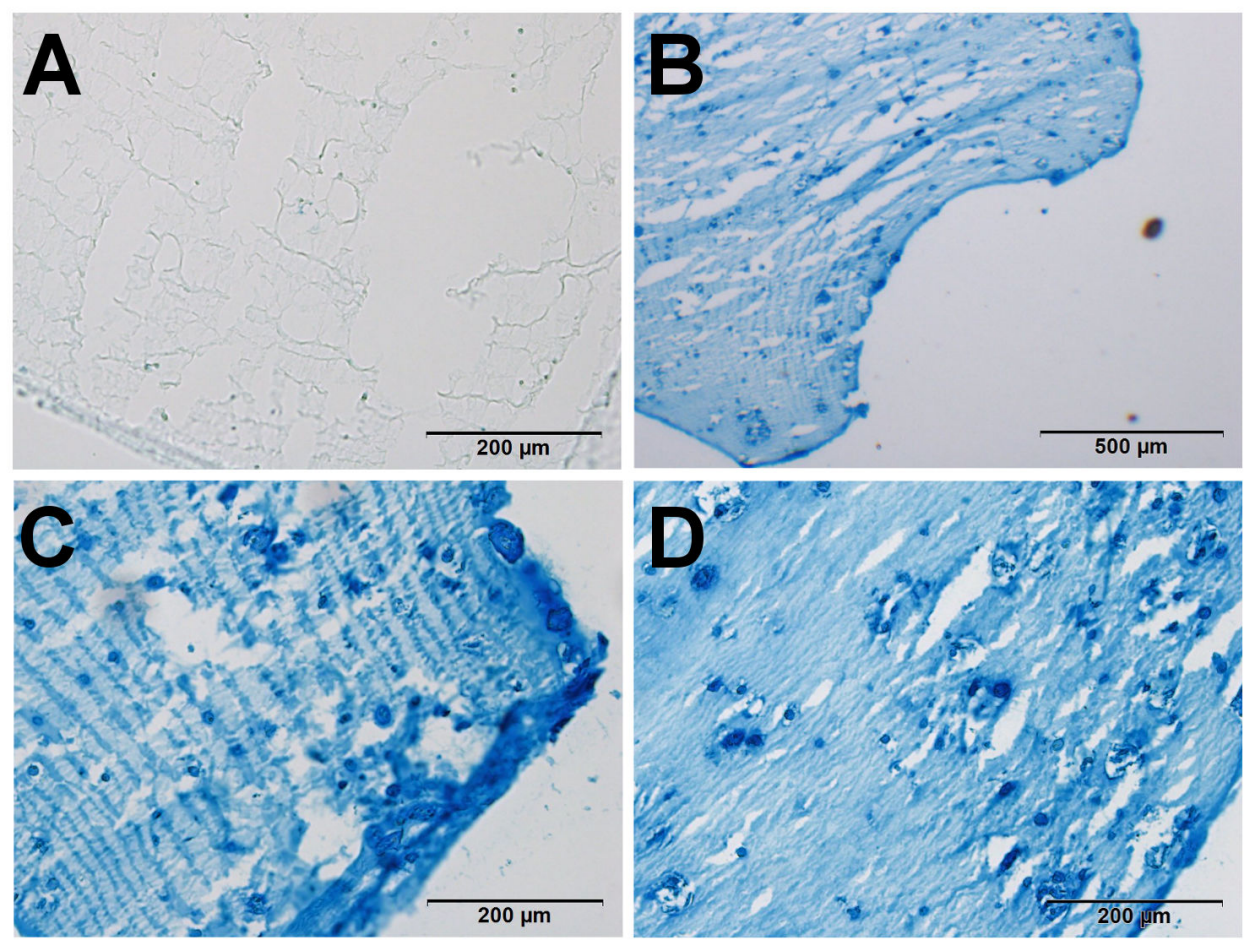
Proteoglycan visualization in alcian blue staining of alginate beads. Freshly prepared beads (A) only have slight background staining of alcian blue, almost all proteoglycans are removed from the cells during the digestion process. In the overview after serum preculture and five weeks of culture (B), the cells impose as round, singular cells with surrounding deep-blue pericellular matrix and further removed matrix in the gel. No differences can be visualized in the GAG content between T3-treated (C) and Dex-treated (D) chondrocytes.

Alcian Blue staining of cryosectioned alginate beads for glycosaminoglycans was completed to confirm findings of biochemical analysis. After five weeks of culture, cells demonstrate a typical chondrogenic phenotype with a darker stained ring of pericellular matrix around each cell. In histology, no difference was seen between the Dex and T3 group, and both showed intense uptake of alcian blue (Figure 8).

**Figure 9:**
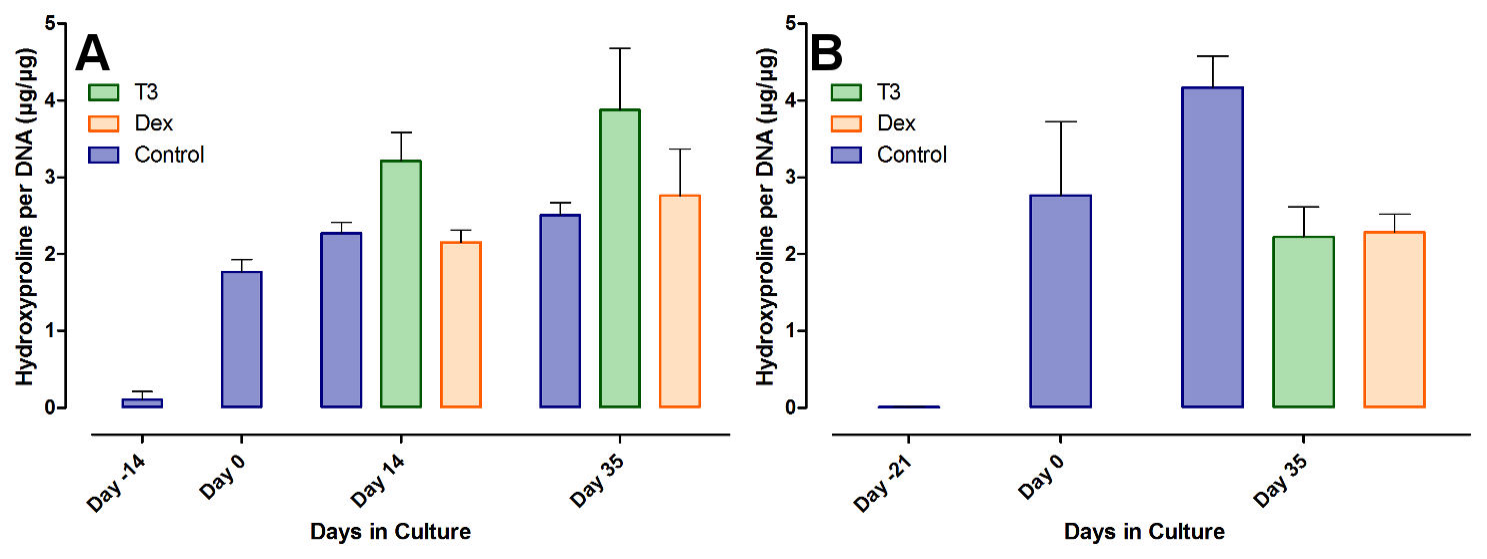
Hydroxyproline production in chondrocytes. Total amount of HYP was quantified and calibrated to the total amount of DNA, to equalize for production merely due to increased or decreased cell number. Shown are the results of the quantification in the articular chondrocytes (A) and the MSCs (B) before and during hormonal treatment, as mean +/- SD.

To determine whether the changes in the mRNA expression actually lead to differences in collagen production levels, total hydroxyproline per DNA was measured in the samples. The T_3_ group in the chondrocytes seems to show an increase over control and Dex after 14 and 35 days, an effect that was not seen in the MSCs (see Figure 9). During chondrogenesis of the MSCs, the cells produced significant amounts of HYP (as already shown in Figure 2), but in both T_3_ and Dex groups this did not increase any further afterwards. Only in the control group did the content continue to rise. Except for the statistically significant increase from day -21 to day 0 (P < 0.0001), none of the changes seen were statistically significant.

**Figure 10:**
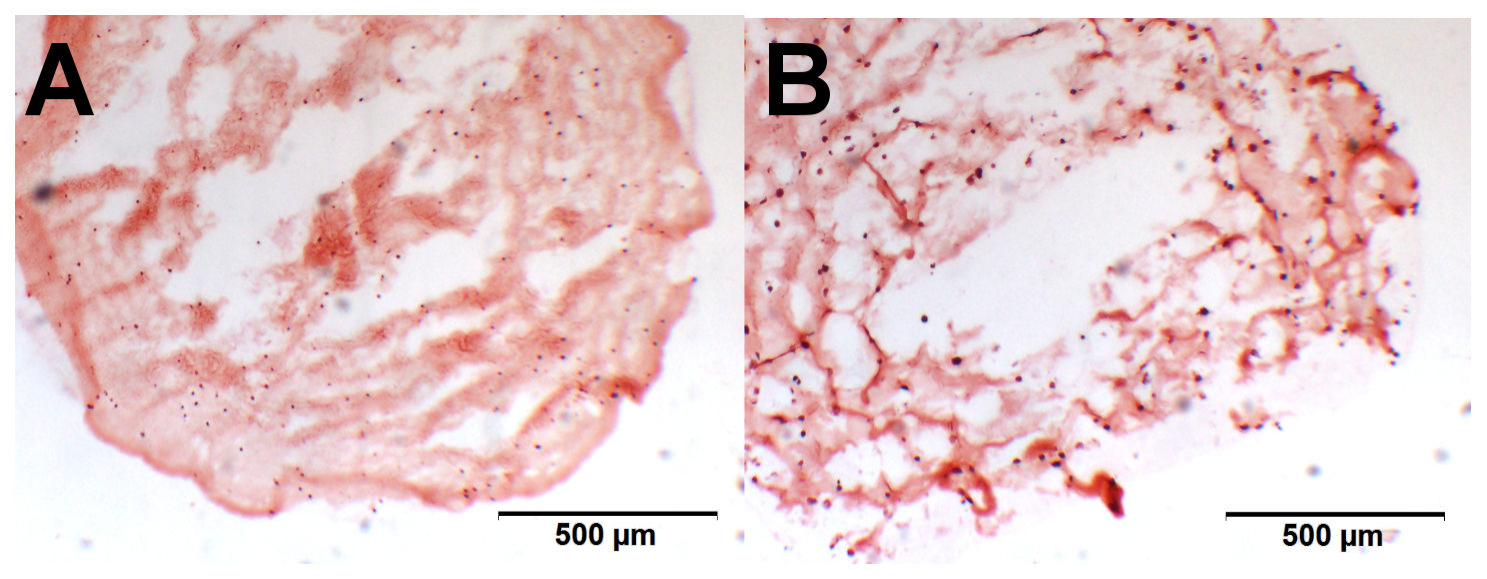
Alizarin red background stain in alginate beads. Freshly prepared beads, stained with AR with (A) representing a bead seeded with chondrocytes and (B) a bead with MSCs. Both show considerable high but very similar background staining, due to the use of CaCl_2_ for crosslinking. The calcium ions remain crosslinked in the alginate throughout the culture period.

The alizarin red staining of samples to investigate calcification led to a degree of background staining due to the calcium present to gelate the alginate. In our hands, this background staining with alizarin red was present on freshly prepared and cultured beads alike (see Figure 10).

**Figure 11:**
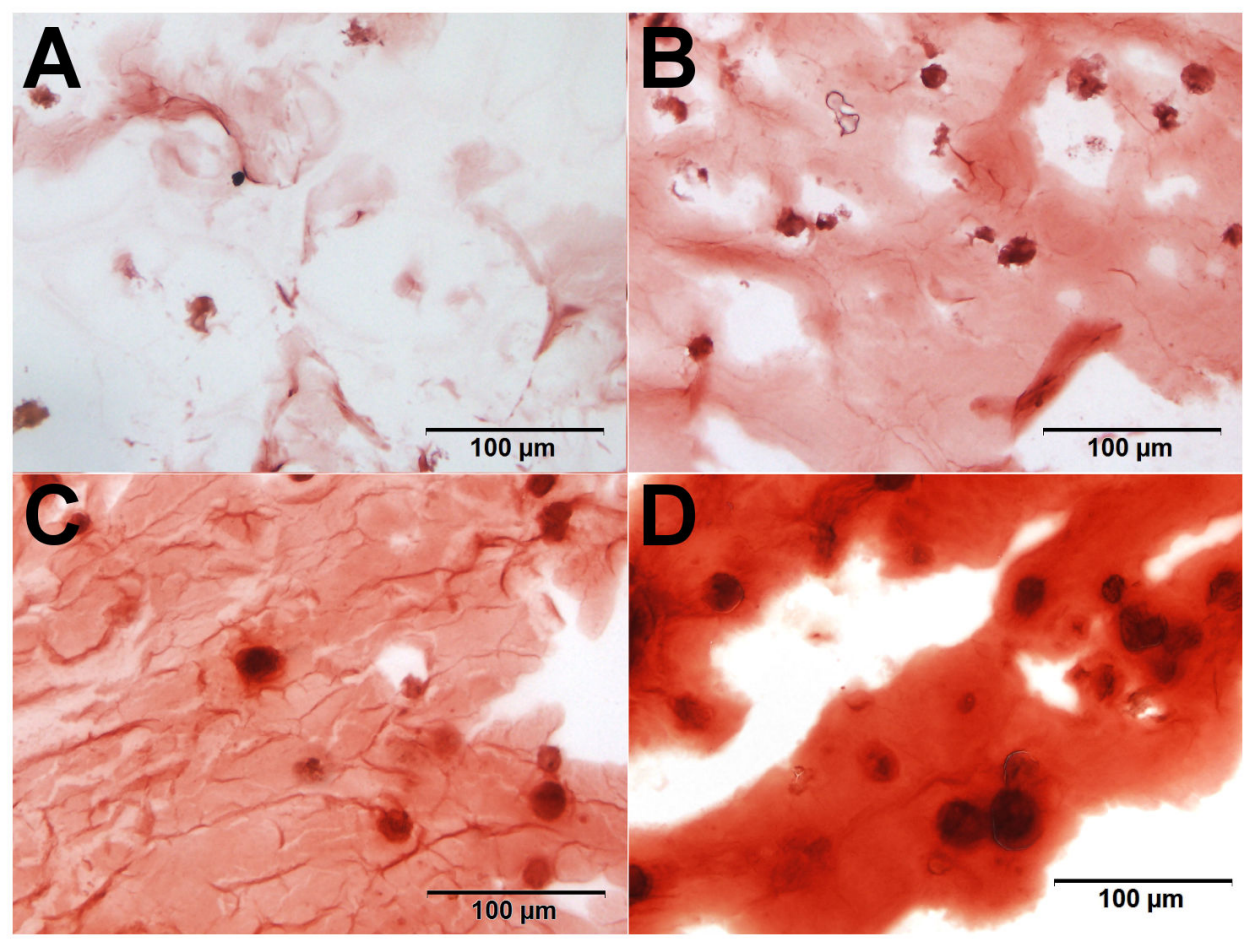
Calcium incorporation in alginate beads with MSCs. For MSC beads, we found an increase in the uptake of AR from day -21 (A) before differentiation and day 0 (B). The Dex-treated beads then showed a much more intense staining, and at higher magnification, the Dex-treated cells (D) showed a pericellular matrix and dark red calcium depositions around the cells, which was not seen at this intensity in the other, T3-treated samples (C).

**Figure 12:**
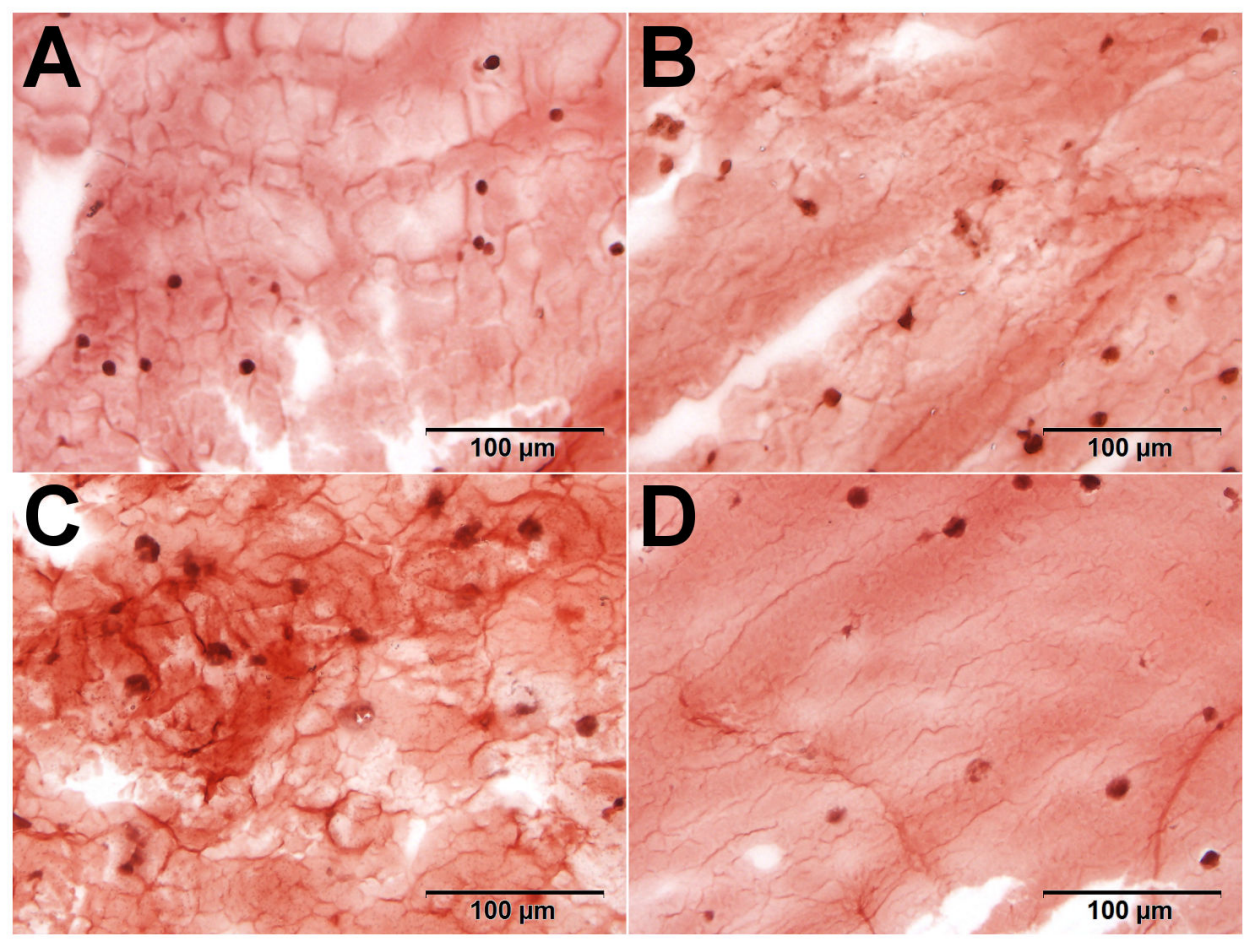
Calcium incorporation in alginate beads with chondrocytes. Between day -14 (A) and day 0 (B) no major increase was seen in AR uptake. Chondrocyte seeded beads treated with T3 (C) showed then a more heterogeneous stain of AR after induction, as compared to the dex-treated group (D).

Despite the backgroup staining observed, differences could be detected. For MSC beads, an increase in the AR staining intensity was observed from day -21 and day 0. The Dex-treated beads then showed a much more intense staining, and at higher magnification, the Dex-treated cells showed a pericellular matrix and dark red calcium depositions around the cells, which was not seen at this intensity in the other samples (Figure 11). The same impression was gained from the T3-stained chondrocyte beads, showing a more heterogeneous stain of AR after induction. However, this effect was not very pronounced and evaluation is more subjective (see Figure 12). The results of the AR staining in general reflect the results of ALP quantification.

## Discussion

In this study, we investigated the effects of two hormones, T_3_ and Dex, on chondrocytes and chondrogenically differentiated MSCs in different settings, aiming to determine if and how these hormones would influence cell proliferation, matrix production and the expression of Col I, Col II and Col X mRNA as markers for phenotypic stability. We had hypothesized that the hormones would induce Collagen X expression, ALP protein activity and increased calcification as markers of terminal differentiation. We also expected articular chondrocytes and chondrogenically differentiated MSCs to be similar in their response to the hormonal stimuli.

We have used full thickness cartilage from bovine fetlock joints as a source of articular chondrocytes, and encapsuled the cells into alginate beads. The cells were seeded at a concentration of app. 4*10^6^ cells / ml of alginate, a concentration that is likely to be close to the cell density in human adult knee cartilage [47]. Experiments were conducted on cells of three different animals, with the cells from each animal kept individually at all times. The resulting variance between the different runs of the experiment account for the possible bandwidth of inter-individual stimuli response. Stripping the cells of almost all their matrix is likely to influence the cellular response to any stimulus [48], but is unavoidable during cartilage digest.

Most of the cells acquired in the digestion process were fully differentiated into the articular chondrocyte phenotype, as seen with Col II mRNA being expressed in high amounts and very little Col I mRNA being expressed. Unexpectedly there was considerably high expression of Col X mRNA in the freshly isolated chondrocytes. In young animals, ossification is still occurring in the lower level of articular cartilage where it is directly adjacent to bone. Though care was taken during harvest not to scrape into the mineralizing layer, it is possible cells were taken from the calcifying zone that were expressing both Col X mRNA and ALP activity [49]. A rapid down-regulation in all groups during the first weeks of culture indicates that whatever cells expressing the Col X mRNA either stopped doing so or potentially completed the process of terminal differentiation and underwent apoptosis. Further work would be required to determine which process actually occurred.

The cells readily responded to serum as a medium supplement inducing proliferation and matrix production. In the presence of serum, the cells doubled in number within two weeks, and produced both collagen and proteoglycan as the typical markers of adult chondrocytes. However, we also saw the previously described trend towards a dedifferentiation [50] by way of a rise in Col I mRNA, even though the cells were encapsuled in a three dimensional culture system. For the further study of the specific effects of the selected hormones, FCS was omitted in the induction phase of the experiments. In serum-free medium, proliferation and matrix production are mostly arrested, but non-supplemented ITS medium was sufficient at all times to support the number of chondrocytes initially entrapped in the beads over a culture period of 35 days in our hands as in other studies [51].

This did not hold true for MSCs: Their count decreased significantly when medium was changed from differentiation medium (with TGF-β1 and Dex) to the serum-free medium. The most common and best described method of chondrogenic differentiation of MSCs is that of pellet culture under the influence of TGF-β, resulting in differentiation towards a chondrocyte-like phenotype, characterized by upregulation of cartilage-specific mRNA and cartilage ECM molecules such as collagens and proteoglycans [52]. Apart from the fact that the cells cannot be separated out of these high density cultures after differentiation, these common *in vitro* conditions can also induce features of fibrocartilage (Col I) as well as features typical of hypertrophy (Col X and ALP) [8,53]. The encapsulation of MSCs into alginate beads for differentiation and further use therefore seemed beneficial to us and others in this setting [54]. The expression pattern of Col I, II and X was comparable to those of pellet cultures, and even without TGF-β, cells differentiated chondrogenically in alginate. In alginate culture, the cells had a non-significant increase in Col X mRNA, accompanied by an increase of ALP activity. It was speculated elsewhere that there is no stable arrest in MSCs after differentiation and before hypertrophy [8]. The matrix production in alginate exceeded that of the pellet cultures in means of collagen production or retention, but not in proteoglycan content.

We have treated the entrapped cells with two different agents in a chemically defined medium to investigate the effect of the hormones in regards to cell proliferation, matrix production and collagen expression pattern. Our main issues were phenotypic stability as well as signs of terminal differentiation of the chondrocytes.

Review of the literature shows many studies using T_3_ on chondrocytes of different origin, mostly growth plate and fetal, and in many different culture systems and media conditions. While human articular chondrocytes subjected to T_3_ in monolayer and serum containing medium showed enhanced proliferation [55], earlier studies showed no effect of T_3_ or T_4_ on adult bovine cartilage cell proliferation or GAG synthesis in high-density cultures in serum-free medium [56]. This is consistent with our findings of no difference between T_3_-treated chondrocytes vs. control in means of DNA and GAG content, and in line with other studies performed in serum-free medium [57]. The continuous production of GAGs within the beads also indicates a stable chondrocytic phenotype of the cells, and stains on cryosectioned beads towards the end of the culture period confirmed the deposition of a pericellular matrix around the chondrocytes, as described by others [35]. Obviously, matrix synthesis and cell proliferation are influenced by a vast number of factors, including nutrient supply, cell culture system and seeding density, which potentially overrule the possible stimulation by growth hormone T_3_.

As markers for chondrocyte hypertrophy we have measured Col X mRNA levels, ALP protein activity in the beads and evaluated calcium incorporation histologically. Collagen Type X is considered a marker of chondrocyte hypertrophy and has been frequently used as such in many publications. Along with its appearance in the growth plate, Col X mRNA is also expressed by chondrocytes in osteoarthritic joints [58]. Over the whole culture period, T_3_ generally had an upregulating effect on all types of Col mRNA, including Col X mRNA. While the receptor and pathways of T_3_ induced effects in growth plate chondrocytes could be recently identified [59], this has not been sufficiently studied in articular chondrocytes. By proving that paracrine signaling via PTHrP can stop the hypertrophic effect of T_3_, it seems likely that these pathways, remnants of the growth plate cartilage, are still active in the “permanent” articular chondrocytes as well [60]. In other experiments, T_3_ was found to be stabilizing dedifferentiated human chondrocytes of various sources and inhibit hypertrophy-inducing effects of other agents [61]. Adult porcine articular chondrocytes have a high affinity receptor for T_3_ and show enhanced ALP activity and an increase in Col X production when stimulated in monolayer and serum culture [62].

In our study, T_3_ in the absence of Dex also showed a tendency to induce terminal differentiation in the articular chondrocytes. The increase in Col X mRNA was accompanied by a rise in ALP activity over the control group. Apoptosis is closely associated with terminal chondrocyte differentiation [63], but no decline in DNA content was detected in our experiments, and no screening test for apoptosis was completed. Possibly, terminal differentiation does not progress to the final apoptotic step within the culture period applied. It has also been suggested that hypertrophy and apoptosis might not necessarily be linked, but regulated along different pathways [64]. We also failed to show other features of hypertrophy such as significant changes in cell morphology, although the alizarin red staining suggested there was potentially a slight increase in calcium deposition. Therefore it is possible that T_3_ in the absence of Dex is sufficient to promote Collagen X expression in the articular chondrocytes, but does not necessarily induce the full features of the hypertrophic chondrocyte. The differential effect of T3 on collagen type II and col X gene expression in chondrocytes and chondrogenically induced MSCs would suggest that they are not at the same developmental stage. For both collagens, addition of T_3_ lead to an increased expression in chondrocytes compared to control, which was not seen in chondrogenically induced MSCs.

The facts currently known about an effect of T_3_ on chondrogenically differentiated MSCs are very sparse, but evidence suggests that removal of TGF-β, reduction of Dex and addition of T_3_ should induce hypertrophy in the chondrogenically differentiated MSCs at least in pellet culture [65]. Here is should be noted, these experiments were performed in the complete absence of Dex and this might explain the differences seen with other studies. Other authors have suggested a dose-dependent effect of T_3,_ promoting MSC osteogenic differentiation at 10 pM [66]. We had hypothesized that a chondrogenically differentiated MSC, thereby becoming a chondrocyte-like cell, would react like a chondrocyte, which it did not. T_3_ had little effect at all on the MSCs, the only significant difference was a marked and steady decrease in Col II mRNA, while Col I and X mRNA levels remained stable. Indeed, if T_3_ is suitable to promote a more osteogenic phenotype in MSCs, this would be in line with the some of the evidence available so far. The dose used in our experiments was identical for chondrocytes and MSCs, and therefore 100-fold higher than the dose used before to induce hypertrophy in pellet cultures – on the other hand, the use of Dex in those experiments might also be a key factor, as discussed further below.

It is striking that especially the differentiated MSCs of the control group in serum-free ITS-supplemented medium do seem to remain in their chondrocyte-like phenotype over five weeks, with no significant increase of ALP protein activity and no increase in Col X mRNA expression. Alginate as the chosen scaffold material might also have a certain influence to this fact. Alginate binds divalent calcium ions, producing a positive charge within the gel, a feature that seems beneficent to chondrogenesis, as surface chemistries promoting a rounded cell morphology in general seem to do [67]. On the downside this arrest essentially stops all matrix production, for without further supplementation, the production of GAG within the beads is rather low, and there is a decrease in DNA as a sign of cell death and arrest of proliferation.

The second hormone investigated in this study was dexamethasone. As with T_3_, the effect of Dex on the growth plate is a matter of ongoing research, but without doubt, glucocorticoids such as Dex have a major role in the development of the growing skeleton, both through systemic [68] as well as local effects on the growth plate [69]. Besides their clinical use on inflamed joints, GCs find frequent use on articular and other chondrocytes in culture, with their effect on cell proliferation, matrix synthesis and gene expression being dependent on cell source, culture system, cell density and dose of GCs used.

Looking at cell survival, no significant differences between the Dex-treated alginate beads versus control or T_3_-treated beads was observed. While some studies describe a decreased cell survival or increase of apoptosis in chondrocytes submitted to Dex or other steroids *in vitro* [22,70] and *in vivo* [21], others find a protective effect of Dex against apoptosis induced by other factors such as mechanical loading [71]. We have not completed stains or assays to test for apoptosis, and DNA content alone is not a sufficient analysis to out rule apoptotic processes in the beads, but again, microscopic imaging did not show signs of such.

Dex is capable of regulating both degrading and matrix-building components of the ECM in a dose-dependent manner. In the current literature, numerous, partly conflicting results have been reported [72,73]. We found that the Dex-treated chondrocytes produced more matrix significantly faster than the control group or the T_3_ treated group. Such an increase in GAG production was seen by others previously [74]. We postulate that many of the catabolic effects on proteoglycan synthesis seen elsewhere relate to the use of co-factors such as TGF-β, while for Dex alone in a chemically defined medium, the anabolic effects on glycosaminoglycan production can be demonstrated. Still, we can also confirm the often-quoted [75] downregulation of Col Type II mRNA. Along with it, we also report a general downregulation of all types of Collagen mRNA measured, most significantly Col I mRNA. If Dex can be used to negate the dedifferentiating influences of other agents, as suggested by the decreasing Col I mRNA levels, it could be an issue for future investigations. Notably, trends of mRNA expression seen in the initial phase of cell culture rarely continued during the later phase of the experiment. Expression levels either stabilized or changed after two or three weeks in culture, stressing the importance of long term experiments as well as regular time points to monitor cellular response. The initial downward trend in collagen expression never reversed in the Dex-treated group, and continued throughout the culture period for Col II mRNA. In long term cultures, this may destabilize the ECM.

Apart from its previously debated apoptotic effect, Dex is also under debate as an inductor of chondrocyte terminal differentiation. Addition of Dex, to monolayer human chondrocytes in serum supplemented culture increased hypertrophy and apoptosis [76], but not in a serum-free environment [26]. In the course of our experiments, investigating Col X mRNA, ALP activity and cell morphology, we have found no evidence of Dex inducing the bovine articular chondrocytes to terminal differentiation. To our knowledge, there is no study on adult articular chondrocytes in a 3D-environment proving otherwise. On the matter of Dex and undifferentiated MSCs, the issue seems to be much clearer. Dex is an inherent part in the osteogenic differentiation of MSCs, and also included in the formulations of chondrogenic and adipogenic differentiation media, as we have also used it for the differentiation in this study. We saw that the chondrocyte-like cells differentiated from MSCs did not benefit from a further supplementation of Dex in the medium in means of cell survival. With the withdrawal of TGF-β1 after successful chondrogenic induction, DNA quantities dropped significantly with or without Dex. The cells were indeed chondrocyte-like in means of GAG production, which was also enhanced by Dex in the serum.

Phenotypic stability is a major issue with MSCs in tissue engineering, especially when aiming for a cartilage repair graft. For this reason, differentiated MSCs must be committed to their cartilage-like gene expression pattern, also in the absence of chondrogenic factors or in the presence of osteogenic factors, a feature that seems to be created even through a short TGF-β exposure [77]. The absence of TGF-β did not cause a loss of cartilage-like gene expression pattern in our hand, but the addition of Dex, as an osteogenic factor, did. Col II mRNA remained stable, but Col X mRNA did increase considerably and steadily with Dex in the medium. Others have discovered similar effects, for example was it shown for human MSCs in three dimensional alginate bead culture to express Col X, after only one week of treatment in Dex and TGF-β1 containing medium [20]. It seems that this process is not interrupted through priming to the chondrogenic phenotype with TGF-β. While we also saw the up-regulation of the chondrogenic gene Col II during *in vitro* induction of chondrogenesis, the differentiating cells also exhibited Col X as a marker of hypertrophy. The phenotype of MSCs in cartilage repair can therefore be so unstable that differentiation will continue along the endochondral ossification pathway, especially with Dex present in the medium [67]. While unfavorable for cartilage, this gives rise the possibility to engineer MSC-based bone grafts, recapitulating the processes of endochondral ossification, acknowledging the “paradigm shift” of developmental engineering [78].

In summary, chondrocytes are capable of more than merely synthesizing cartilage matrix. Their terminal differentiation is an important process in long bone growth and secondary fracture healing. It is also suspected that it plays a role in onset of degenerative OA, yet the factors that influence it are poorly understood. Also, chondrocytes from various sources show different behavior towards their response to any stimuli. Without defined conditions, a comparison of completed studies is without benefit. Currently, too little work is completed on primary adult chondrocytes to lead to later clinical applications. In this study we have taken a step towards probing T_3_ as an inductor of chondrocyte hypertrophy in postnatal immature articular chondrocytes. We have also shown a contrary effect of Dex to contribute to the ongoing discussion of the use of this hormone in cartilage tissue engineering. We also want to suggest the idea that even those chondrocytes deemed “permanent” may not be so, but rather be “slow transient” in their response to stimuli, a fact that must be considered when using these cells in tissue engineering applications.

The use of MSCs in bone and cartilage tissue engineering is well established, but phenotypic stability is a major issue, especially when aiming for a cartilage repair graft. It remains under debate whether or not the phenotype of the chondrocyte-like cell after differentiation is stable enough to be used in cartilage repair. When MSCs are indeed moving on towards terminal differentiation after chondrogenic differentiation, they can be used to generate a model of endochondral ossification. We can contribute with our results, recommending the use of alginate beads rather than micromass pellet cultures as a scaffold, and the use of Dex to trigger Col X mRNA expression in these cells.
